# Supplementary material for: Sexual selection theory meets disease vector control: Testing harmonic convergence as a “good genes” signal in Aedes aegypti mosquitoes
Source: PLoS Negl Trop Dis. 2021 Jul 2;15(7):e0009540. doi: 10.1371/journal.pntd.0009540 (PMC8282061; doi:10.1371/journal.pntd.0009540)
Supplement: S1 Text — (DOCX) [file pntd.0009540.s001.docx]

**Supporting information:**

**Methods**

***Parental rearing***

Mosquito eggs were vacuum hatched for 20 min and received a pinch of powdered fish food diet (Hikari Cichlid Gold, Hayward, CA, USA). Hatched larvae were held at 27°C and 85% relative humidity (RH) overnight. The following day larvae were sorted into groups of 200 and placed in 1 L of distilled water in plastic trays (28 x 21 x 8 cm) to obtain medium body sizes [1]. Each tray was provided with four large pellets of fish food and monitored daily. Fitness differences often remain undetected under optimal rearing conditions, and environmental stress has been shown to be important for measuring genetic benefits in many animals [2–4]. Because we previously found that differences between offspring of converged and non-converged males are detectable only after application of a moderate level of temperature stress [5], we therefore exposed 4^th^ instar larvae to 21ºC ambient temperatures for 24 h. Resulting pupae were individually placed in 15 mL tubes plugged with cotton wool. Upon emergence, males and females were held in sex-specific cages (8 L plastic buckets) where they fed a 10% sugar solution *ad libitum*.

In all experiments, mosquito wing lengths were obtained from a subset of mosquitoes from each experimental group as a proxy for overall body size as previously described [6]. For reproductive fitness experiments, regardless of whether parent or offspring data were used in subsequent analyses, father (N=90) and mother (N=84) wing lengths were 2.23 ± 0.10 and 2.96 ± 0.12 mm, respectively, and son (N=72) and daughter (N=139) wing lengths were 2.42 ± 0.34 and 2.93 ± 0.10 mm, respectively. For flight performance experiments, father (N=34) wing lengths were 2.13 ± 0.13 mm.

***Harmonic convergence assay***

Females were tethered using the “semi-tethering” technique to optimize flight mobility while maintaining female proximity to the microphone as previously described [5,7,8]. Briefly, females were tethered at the dorsal mesothorax by a human hair attached to an insect pin using nail glue (L.A. Colors, Ontario, CA, USA or similar product). Tethered females were placed approximately 3 cm away from a particle velocity microphone (NR-21358; Knowles Electronics, Itasca, IL, USA) attached to a custom amplifier [9] in a plastic recording arena (20 x 14 x 10 cm) [10]. Female flight was stimulated by using an aspirator to release females from tarsal inhibition or to deliver brief pulses of air [5,8].

Potential instances of harmonic convergence were systematically tested at all harmonic combinations below 3,000 Hz, including at the female second and male first harmonics (2:1), the female third and male second harmonics (3:2), the female fourth and male third harmonics (4:3), the female fifth and male third harmonics (5:3), and the female fifth and male fourth harmonics (5:4) [11]. To be considered a true instance of convergence, the harmonics had to be within 5 Hz of each other for a duration of at least 1 s [5]. If no courtship flight interactions occurred within 5 min, we considered it a non-event and started a new trial with a fresh set of mosquitoes.

***Offspring generation and rearing***

*Offspring reproductive fitness assays*

Eggs from females that took blood meals and survived to lay were collected from each mating pair, vacuum hatched, and reared separately. Larvae were reared in plastic cups containing approximately 25 larvae per family and 100 mL of deionized water and 0.05 g of ground fish food pellet. No more than 20 male pupae were placed directly into 0.5 L wax containers supplied with 10% sugar-soaked pads. Female daughter pupae were placed in individual 15 mL tubes. Females used in male insemination capacity experiments and males used to inseminate daughters for the lifetime reproduction study were reared from a separate cohort of unrelated Thai colony mosquitoes as described above for parents.

*Offspring flight performance assays*

For son flight performance assays, 20 larvae per family were held in 100 mL of water and larvae were fed diet *ad libitum.* Offspring pupae were placed into individual 15 mL tubes for adult eclosion. Upon eclosion, offspring were separated by sex and family. Female offspring were discarded while males were sorted into 0.5 L cups by emergence day and family and supplied with 10% sugar.

***Reproductive fitness assays***

*Male fertility: Insemination capacity*

In the case of fathers, the insemination capacity assay followed the initial 24 h parental mating period, in which the father was held with the original female. Each male was then held in an 0.5 L container with five virgin females for 48 h intervals. The spermathecae of females replaced at each interval were dissected and examined for the presence or absence of sperm at the end of each 48 h interval.

*Female fecundity: Eggs laid*

Upon emergence, individual daughters were transferred into 20 mL cartons and held for three days to allow for reproductive maturation. Daughters were then provided three unrelated colony males for 24 h to allow for insemination. After removal of males, females were offered a blood meal (co-author SAP) every three days for the remainder of their lifetime up to 20 days. Oviposition cups with water were added to each carton three days post-blood meal. Females that did not ingest the initial blood meal were discarded and data were recorded on whether females blood fed during each subsequent host offering. Consistent with feeding behavior of *Ae. aegypti* in nature [12,13], females were not provided sugar for the duration of the fecundity assay.

***Flight performance assay***

*Male flight performance: Mating attempts, contacts, and contact success rate*

For the custom-built flight response cage, the speaker (Sony MDR-E9LPL 13.5 mm speaker; frequency: 18-22,000 Hz; sensitivity: 104 dB/mW) was suspended 8 cm below the lid of a 30 cm^3^ clear plastic cage and was attached to a timing belt (Synchroflex Timing Belt, 4660-950, Technobots, Rugby, UK) on top of the cage through a 0.5 x 22 cm track in the lid. The timing belt was driven in a back-and-forth motion across the cage using a small stepper motor (ST411, 18M1804A, Nanotec, Munich, Germany) powered by an enclosed power supply (LRS-150F-12RS, Mean Well Enterprises, Taipei, Taiwan). The entire apparatus of was placed on top of a hot plate set to 28˚C with a worn black t-shirt (co-author LJC) stretched across the top to provide additional visual and olfactory mating cues for males, as males tend to swarm around human hosts when seeking mates [14] and prefer dark colors [15].

Individual males were released into the cage with a silent, immobile speaker to allow for a 15–20 s cage acclimation period in the absence of acoustic stimuli or speaker movement. After this, the silent speaker began to move for an approximately 10 s acclimation period, with the arm taking 15 ms to accelerate to its 0.458 m/s (i.e., 0.48 s to move across the 22 cm track) back-and-forth speed across the cage. Finally, audio playback began and continued for two minutes during the moving playback period, where a 550 Hz pure tone playback was presented at a volume of approximately 60 dB in 10 s bursts with 5 s of silence in between. After the trial, males were removed from the flight response cage and held individually in 30 mL cartons at 27˚C and 80% RH overnight before testing in harmonic convergences assays.

Acoustic stimuli most likely to attract free-flying males were identified through a series of pilot playback experiments [16]. The microphones and ear bud speaker were placed in the center of a 20 cm^3^ cage and secured on top of a polystyrene platform (13 x 10 x 5 cm). A particle velocity microphone was placed 0.5 cm below the speaker and a pressure microphone (FG-23329-C05; Knowles Electronics, Itasca, IL, USA) was placed adjacent to this. For each trial, groups of 15 males were randomly collected from the colony cage and released into the flight cage. There was a 30 s period for the males to acclimatize before the playback began. The two-minute playback consisted of 15 s of pre-stimuli silence with three bursts of 15 s stimuli with 20 s of rest in-between each burst. For any one cage, each of the six frequency treatments were played twice in a randomized order, giving 12 recordings per cage. We recorded the number of males approaching a given frequency in each trial cage. This experiment was performed twice giving a total of 28 trials with 420 males. We identified 550 Hz as the frequency that most free-flying males responded to [16].

A second pilot experiment was conducted to determine at what speed the movement of the speaker became discerning. Five males per trial were released into the flight response cage. The speaker was driven at 200, 300, 400, and 500 RPM. A 550 Hz tone was played for 10 s pulses with a 5 s rest period in between each pulse for a total of two minutes. Video of each trial was recorded using a stationary HD camera positioned along the same horizontal axis as the speaker movement. We recorded the number of attempts made by the five males and the number of males able to contact the microphone during this trial period. We replicated this assay twice for a total of three experiments per speed (5 males/speed/experiment). Males were most responsive to a speaker moving at 300 RPM. On average, there were 8.67 ± 3.38 mating attempts with 30.54% of these including a contact with the speaker. By contrast, the contact success rate for a stationary speaker playing the same stimuli is 100% and a speaker moving at 400 RPM is only 3.3%. While some mating attempts were made at all speeds, no males in the 200 and 500 RPM groups were successful at contacting the speaker.

***Statistical methods***

*Distribution and variability of reproductive fitness and flight performance traits*

To characterize the distribution and variability of parent and offspring reproductive fitness and flight performance parameters, data were first tested for normality using a one-sample Kolmogorov-Smirnov (KS) test in SPSS. Distributions were then compared for equality using an independent samples Kruskal–Wallis (KW) test. Descriptive statistics of fitness and performance data were produced to determine skewness, standard deviation, and other dispersion statistics (S2 Table).

*Heritability of reproductive fitness and flight performances traits*

To assess the heritability of the reproductive fitness and flight performances metrics, we ran linear mixed effects models (LMMs) [17,18] using a gaussian distribution to test for relationships between parental and offspring traits. In each case, the parental trait (father insemination capacity, father wing length, number of eggs in mother’s first clutch, or mother wing length) and experimental trial were incorporated as fixed effects and family was incorporated as a random effect to account for the measurement of multiple offspring from a single parental pair. Fixed effects were tested using F tests with a Satterthwaite approximation for the denominator degrees of freedom and assumptions of normality and homogeneous variances were checked by visually assessing residuals. For reproductive fitness assays in sons, we assessed the effect of parental traits on son insemination capacity and wing length response variables. For reproductive fitness in daughters, we assessed the effect of parental traits on daughter eggs laid in first clutch, total eggs laid, eggs laid per blood meal, days alive, and wing length response variables. For flight performance assays, we used the same approach to test the relationship between father and son flight metrics, including total mating attempts, total contacts, and contact success rates using LMMs, and probability of contact using a generalized linear mixed model (GZLMM) [17].

*Harmonic convergences signaling of parent and offspring inherent quality*

To test whether harmonic convergence is used as an acoustic signal of reproductive fitness and inherent genetic quality, we first determined whether convergence status predicted fitness traits within the parental generation. Linear models (LM) [17] with harmonic convergence status, experimental trial, and wing length incorporated as fixed effects were used to test for differences in father insemination capacity and mother eggs laid in first clutch response variables. For LMs, fixed effects were tested using F-tests and assumptions of normality and homogeneous variances were tested as with LMMs. A LM with harmonic convergence status, experimental trial, and mating status as fixed effects was used to test the effect of convergence on father total attempts, total contacts, and contact success rate response variables. A generalized linear model (GZLM) [17] with a binomial distribution and the same effects was used to assess the impact of convergence status on the probability that the father successfully contacted the speaker at least once in the trial. For GZLMs, fixed effects were tested using t-tests and z-tests.

Next, to examine whether harmonic convergence cues serve as indicators of indirect effects on offspring fitness, we tested whether parental convergence status predicted offspring fitness measures. For sons, LMMs [19] with parental convergence status, experimental trial, and wing length as fixed effects and family as a random effect were used to test the effect of parental convergence on son insemination capacity (response variable). We also tested whether son convergence status predicted son insemination capacities using the same models. We used LMMs [17] to assess whether the fixed effects of parental convergence status and experimental trial or the random effect of family had an effect on son total mating attempts, total contacts, contact success rate, or probability of contact response variables. Here, as before, a gaussian distribution was used in all LMMs, except probability of speaker contact data (GZLMM), which used a binomial distribution. For GZLMMs, fixed effects were tested using z-tests or likelihood ratio chi-square tests (aka deviance tests). To test for an association between parental and son convergence status (response variable), we also used a GZLMM with a binomial distribution, experimental trial as a fixed effect, and family as a random effect.

For daughters, LMMs [19] with parental convergence status and experimental trial as fixed effects and family as a random effect were used to assess the effect of parental convergence on daughter eggs laid in first clutch, total eggs laid, eggs laid per blood meal, and days alive response variables. Kaplan-Meier curves were used to visually compare daughter survival based on parental convergence status [20–22]. A Cox frailty model was used to test for differences in survival between the curves while controlling for experimental trial as a fixed effect, cumulative blood meals as a time-varying fixed effect, and family as a random effect [23,24]. We additionally used the individual day level data to construct a daughter life table [25–28]. Using these data, we calculated the intrinsic rate of increase (*r*)*,* cumulative reproductive rate (*R_0_*), and generation time (*T_c_*) for offspring of converged and non-converged parents from the two life table experimental trials.

**Results**

***Females that mate with higher quality males do not produce higher quality offspring***

In the case of male fertility, neither father (KS test, P<0.001) nor son (P=0.002) insemination capacities were normally distributed, with the former skewing strongly left (-1.874) and the latter skewing moderately right (0.824; S2 Fig A and B and S2 Table). Although fathers inseminated approximately 26% of females presented compared to 28% for sons, their insemination capacity distributions did not differ (KW test, P=0.753). However, insemination capacity did vary moderately within generations, with individual males ranging from as little as 0 or 15% insemination rates to over 50% (S2 Table). Although father and son size as determined by wing length differed significantly in their distributions (KW test, P=0.041), fathers (2.21 ± 0.09 mm) and sons (2.16 ± 0.10 mm) were of comparable size and varied only moderately (S5 Fig A and B and S3 Table).

With respect to female fecundity, neither mother (KS test, P=0.008) nor daughter (P<0.001) first egg clutch sizes were normally distributed, with both the former (0.275) and latter (0.103) skewing weakly right (S2 Fig C and D and S2 Table). Consistent with previous observations in low and high generation colonies [38,40,62], both mother (SD=78.367) and daughter (35.984) first egg clutch size distributions were extremely variable (S2 Table) and differed significantly (KW test, P<0.001), perhaps due in part to constraints associated with using a low generation population of mosquitoes recently collected from the field. Daughters laid 57.66 ± 35.98 eggs, roughly 54% fewer eggs than mothers, who laid 124.15 ± 78.37 eggs. As with fathers and sons, although mother and daughter size distributions differed (KW test, P=0.004), mothers (2.98 ± 0.10 mm) and daughters (2.93 ± 0.10 mm) were nevertheless of similar size (S5 Fig C and D and S3 Table). Daughter lifetime fecundity and life history parameters also displayed high levels of variability (S3 Fig and S2 Table). For example, daughter eggs laid per blood meal (SD=28.666) and total eggs laid over her lifetime (116.439) varied remarkably, with the latter ranging from 0–564 eggs (S2 Table).

With respect to male flight performance, neither father (KS test, P≤0.004 for all traits) nor son (P≤0.003 for all traits) flight metrics were normally distributed (S4 Fig and S2 Table), with both father and son total mating attempts and contacts skewing strongly right (≥1.144 for all traits) and contact success rates skewing weakly right (0.003 and 0.110, respectively). Fathers and sons both attempted to mate approximately four times per trial, contacted the speaker roughly three to four times per mating attempt, and successfully contacted the speaker in approximately two-thirds of their attempts (S2 Table). Flight performance distributions did not differ between fathers and sons for any of the assayed metrics (KW test, P≥0.176 for all traits), showing little variation between, but robust variation within generations (S2 Table). For example, male flight performance varied from little to no activity to approximately 20 mating attempts and 50 to 100 contacts. Together, these reproductive fitness and flight performance trait distributions show that parent and offspring fitness traits display robust standing variation in our field-derived colony, thereby providing ample potential for sexual selection.

**References**

1. Helinski MEH, Harrington LC. Male mating history and body size influence female fecundity and longevity of the dengue vector *Aedes aegypti*. J Med Entomol. 2011;48: 202–211. doi:10.1603/me10071.

2. David P, Bjorksten T, Fowler K, Pomiankowski A. Condition-dependent signalling of genetic variation in stalk-eyed flies. Nature. 2000;406: 186–188. doi:10.1038/35018079.

3. Jia F-Y, Greenfield MD. When are good genes good? Variable outcomes of female choice in wax moths. Proc Biol Sci. 1997;264: 1057–1063. doi:10.1098/rspb.1997.0146.

4. Qvarnström A. Context-dependent genetic benefits from mate choice. Trends Ecol Evol. 2001;16: 5–7. doi:10.1016/s0169-5347(00)02030-9.

5. Cator LJ, Harrington LC. The harmonic convergence of fathers predicts the mating success of sons in *Aedes aegypti*. Anim Behav. 2011;82: 627–633. doi:10.1016/j.anbehav.2011.07.013.

6. Ponlawat A, Harrington LC. Age and body size influence male sperm capacity of the dengue vector *Aedes aegypti* (Diptera: Culicidae). J Med Entomol. 2007;44: 422–426. doi:10.1603/0022-2585(2007)44[422:aabsim]2.0.co;2.

7. Aldersley A, Cator LJ. Female resistance and harmonic convergence influence male mating success in *Aedes aegypti*. Sci Rep. 2019;9: 2145. doi:10.1038/s41598-019-38599-3.

8. Cator LJ, Ng’Habi KR, Hoy RR, Harrington LC. Sizing up a mate: Variation in production and response to acoustic signals in *Anopheles gambiae*. Behav Ecol. 2010;21: 1033–1039. doi:10.1093/beheco/arq087.

9. Arthur BJ, Sunayama-Morita T, Coen P, Murthy M, Stern DL. Multi-channel acoustic recording and automated analysis of *Drosophila* courtship songs. BMC Biol. 2013;11: 11. doi:10.1186/1741-7007-11-11.

10. Villarreal SM, Winokur O, Harrington L. The impact of temperature and body size on fundamental flight tone variation in the mosquito vector *Aedes aegypti* (Diptera: Culicidae): Implications for acoustic lures. J Med Entomol. 2017;54: 1116–1121. doi:10.1093/jme/tjx079.

11. League GP, Baxter LL, Wolfner MF, Harrington LC. Male accessory gland molecules inhibit harmonic convergence in the mosquito *Aedes aegypti*. Curr Biol. 2019;29: R196–R197. doi:10.1016/j.cub.2019.02.005.

12. Edman JD, Strickman D, Kittayapong P, Scott TW. Female *Aedes aegypti* (Diptera: Culicidae) in Thailand rarely feed on sugar. J Med Entomol. 1992;29: 1035–1038. doi:10.1093/jmedent/29.6.1035.

13. Harrington LC, Edman JD, Scott TW. Why do female *Aedes aegypti* (Diptera: Culicidae) feed preferentially and frequently on human blood? J Med Entomol. 2001;38: 411–422. doi:10.1603/0022-2585-38.3.411.

14. Hartberg WK. Observations on the mating behaviour of *Aedes aegypti* in nature. Bull World Health Organ. 1971;45: 847–50.

15. Gilbert IH, Gouck HK. Influence of surface color on mosquito landing rates. J Econ Entomol. 1957;50: 678–680. doi:10.1093/jee/50.5.678.

16. Miller M. Harmonic convergence and attraction in both tethered and free-flying *Aedes aegypti* males, in response to artificial playbacks. M.Sc. Thesis, Imperial College London. 2017. Available: http://hdl.handle.net/10044/1/52400.

17. Bates D, Mächler M, Bolker BM, Walker SC. Fitting linear mixed-effects models using lme4. J Stat Softw. 2015;67: 1–48. doi:10.18637/jss.v067.i01.

18. Singmann H, Bolker B, Westfall J, Aust F, Ben-Shachar MS. afex: Analysis of factorial experiments. R package version 0.27-2. 2020. Available: https://cran.r-project.org/package=afex.

19. Kuznetsova A, Brockhoff PB, Christensen RHB. lmerTest package: Tests in linear mixed effects models. J Stat Softw. 2017;82: 1–26. doi:10.18637/jss.v082.i13.

20. Therneau TM. A package for survival analysis in R. R package version 3.1-12. 2020. Available: https://cran.r-project.org/package=survival.

21. Therneau TM, Grambsch PM. Modeling survival data: Extending the Cox model. New York: Springer; 2000.

22. Kassambara A, Kosinski M, Biecek P. survminer: Drawing survival curves using “ggplot2.” R package version 0.4.6. 2019. Available: https://cran.r-project.org/package=survminer.

23. Therneau TM. coxme: Mixed effects Cox models. R package version 2.2-16. 2020. Available: https://cran.r-project.org/package=coxme.

24. Lenth R. emmeans: Estimated marginal means, aka least-squares means. R package version 1.4.4. 2020. Available: https://cran.r-project.org/package=emmeans.

25. Begon M, Mortimer M, Thompson DJ. Population ecology: A unified study of animals and plants. 3rd ed. Oxford: Blackwell Science; 1996.

26. Villarreal SM, Pitcher S, Helinski MEH, Johnson L, Wolfner MF, Harrington LC. Male contributions during mating increase female survival in the disease vector mosquito *Aedes aegypti*. J Insect Physiol. 2018;108: 1–9. doi:10.1016/j.jinsphys.2018.05.001.

27. Begon M, Townsend CR, Harper JL. Ecology: From individuals to ecosystems. 3rd ed. Oxford: Blackwell Publishing; 1996.

28. Harrington LC, Buonaccorsi JP, Edman JD, Costero A, Kittayapong P, Clark GG, et al. Analysis of survival of young and old *Aedes aegypti* (Diptera: Culicidae) from Puerto Rico and Thailand. J Med Entomol. 2001;38: 537–547. doi:10.1603/0022-2585-38.4.537.
