# Supplementary figures and images for: Sexual selection theory meets disease vector control: Testing harmonic convergence as a “good genes” signal in Aedes aegypti mosquitoes
Source: PLoS Negl Trop Dis. 2021 Jul 2;15(7):e0009540. doi: 10.1371/journal.pntd.0009540 (PMC8282061; doi:10.1371/journal.pntd.0009540)

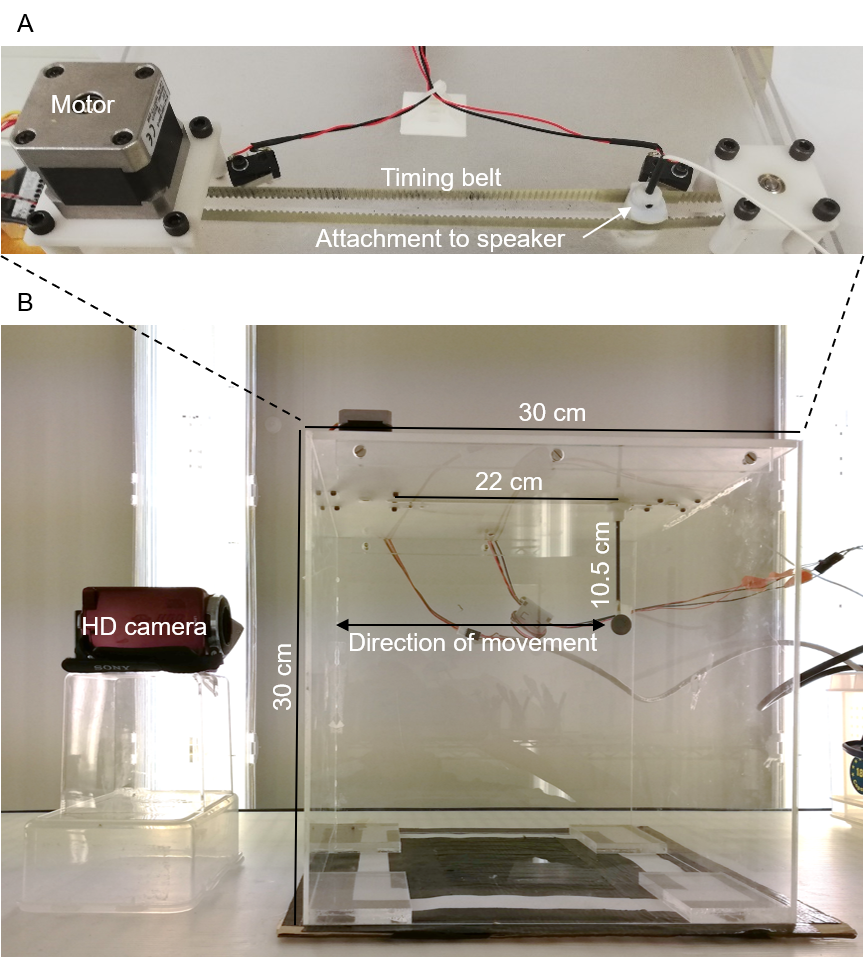

Supplement: S1 Fig — A custom-built flight cage was fit with a moving arm that allowed for playback of audio stimuli from a moving source. A motorized timing belt attached to a speaker (A) was used to move the arm back and forth at fixed speeds. A stationary HD camera was positioned along the same horizontal axis as the speaker movement to capture male responses in the flight cage (B). Photograph credit: LJC. (TIF) [file pntd.0009540.s002.tif]

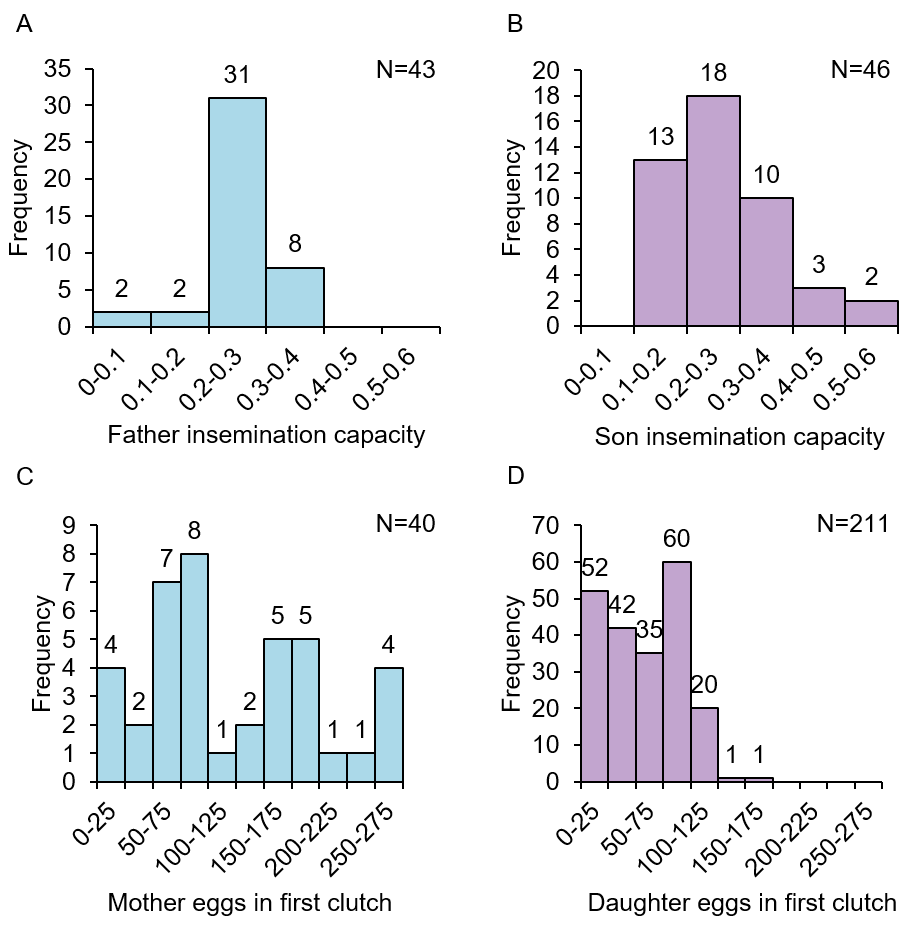

Supplement: S2 Fig — Father and son fertility (A and B) as well as mother and daughter fecundity (C and D) varied substantially. Graphs display distribution sample sizes (N) and the number of samples per bin (above bars). For detailed descriptive statistics, see S2 Table. (TIF) [file pntd.0009540.s003.tif]

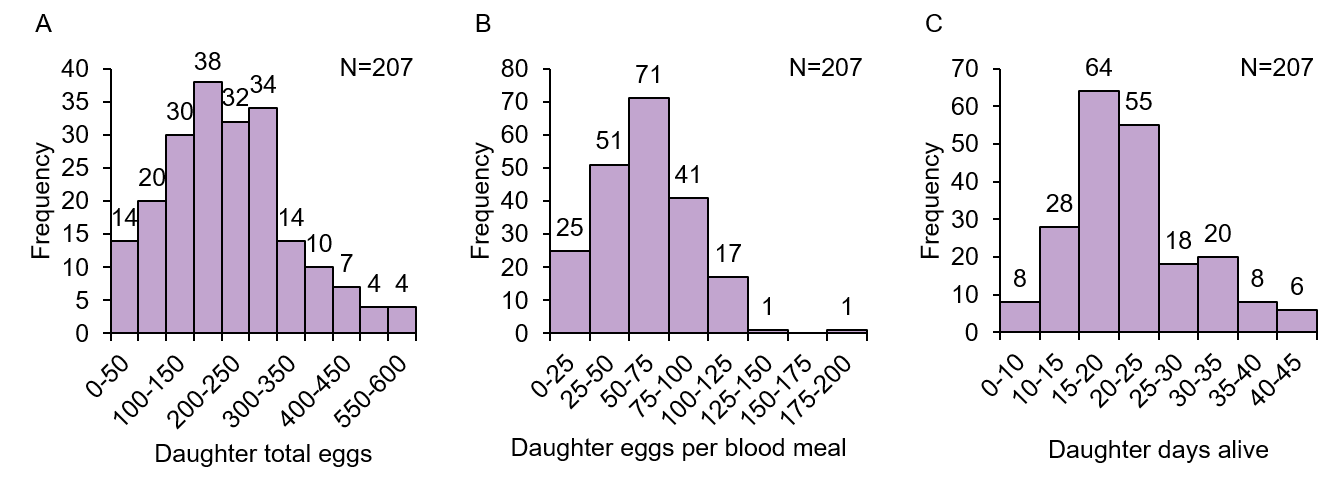

Supplement: S3 Fig — Daughter lifetime fecundity (A), fecundity by blood meal (B), and longevity (C) displayed high levels of variability. Graphs display distribution sample sizes (N) and the number of samples per bin (above bars). For detailed descriptive statistics, see S2 Table. (TIF) [file pntd.0009540.s004.tif]

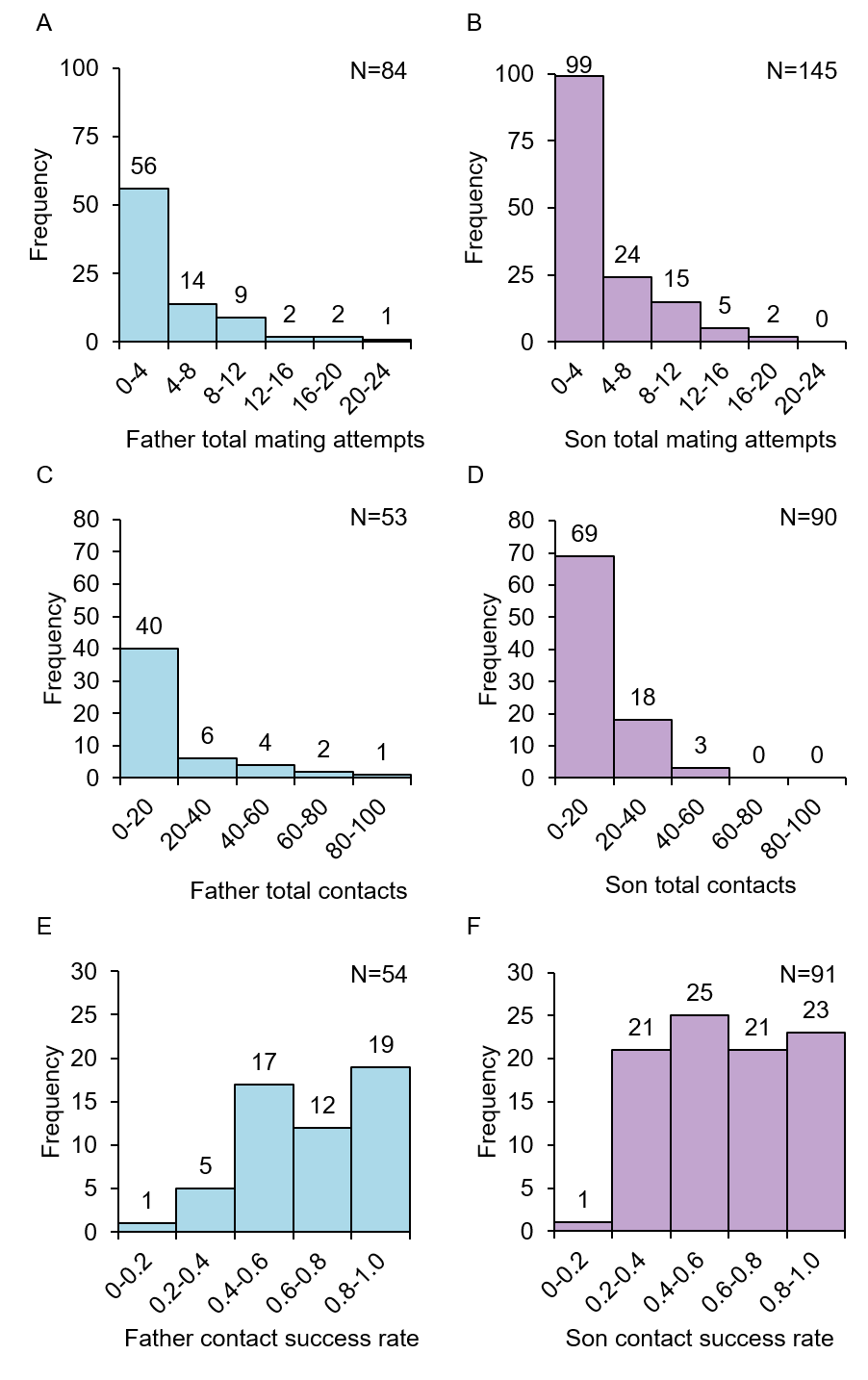

Supplement: S4 Fig — Father and son total mating attempts (A and B), total contacts (C and D), and contact success rates (E and F) displayed strong levels of variation. Graphs display distribution sample sizes (N) and the number of samples per bin (above bars). For detailed descriptive statistics, see S2 Table. (TIF) [file pntd.0009540.s005.tif]

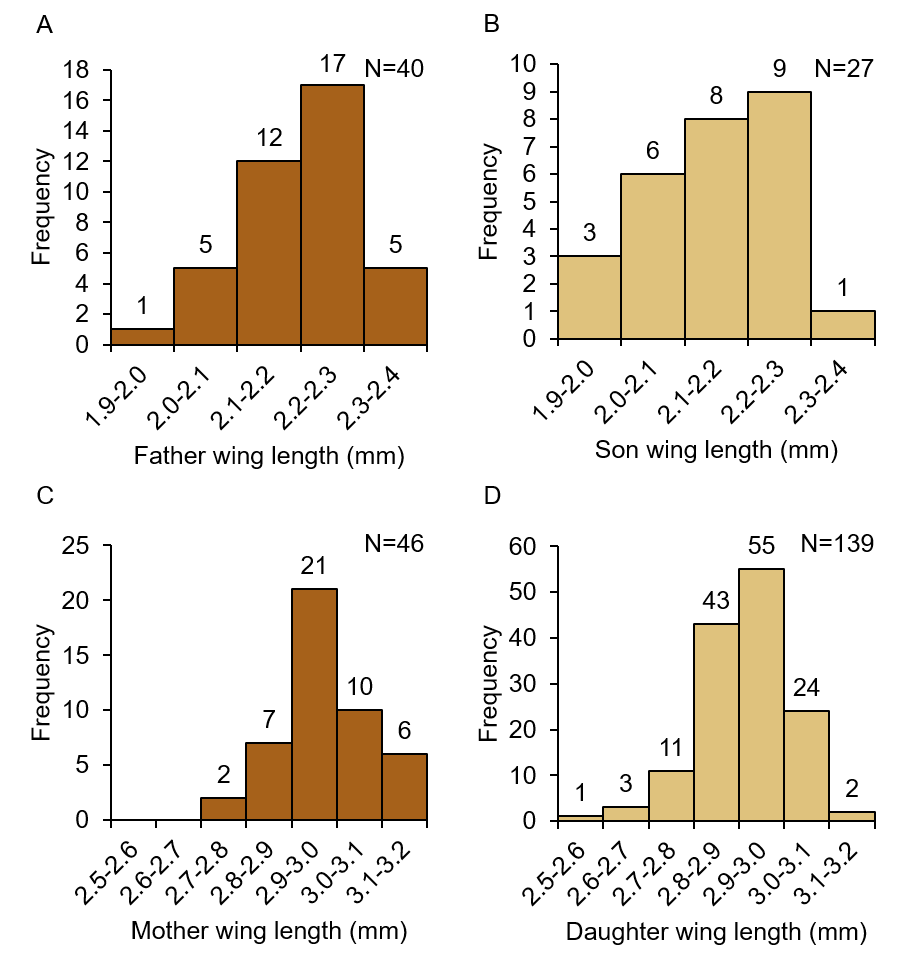

Supplement: S5 Fig — Father and son sizes (A and B) were normally distributed and biologically comparable, despite differing statistically (KW test, P = 0.041). Mother sizes (C), but not daughter sizes (D), were normally distributed and size distributions were biologically similar, despite differing statistically (KW test, P = 0.004). Graphs display distribution sample sizes (N) and the number of samples per bin (above bars). For detailed descriptive statistics, see S3 Table. (TIF) [file pntd.0009540.s006.tif]

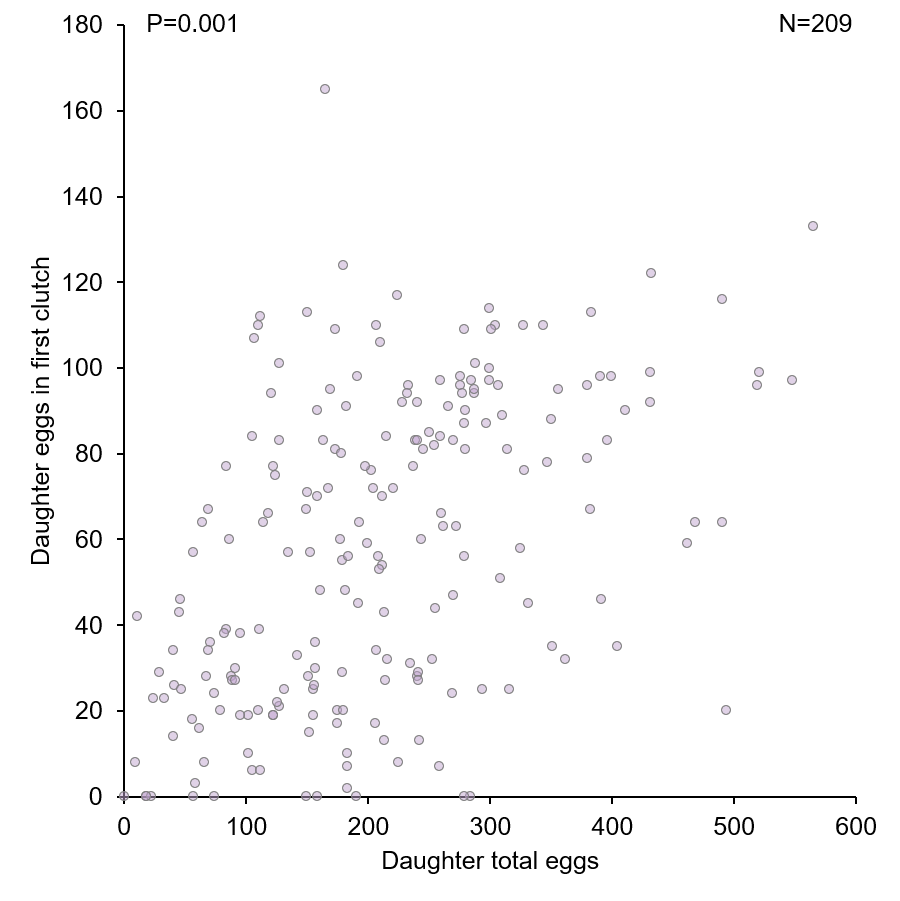

Supplement: S6 Fig — Daughters that laid more eggs in their first clutch tended to lay more eggs across their lifetime (P = 0.001). Graphs display correlation sample size (N) and LMM P-value. (TIF) [file pntd.0009540.s007.tif]
