# Supplementary material for: Sexual selection theory meets disease vector control: Testing harmonic convergence as a “good genes” signal in Aedes aegypti mosquitoes
Source: PLoS Negl Trop Dis. 2021 Jul 2;15(7):e0009540. doi: 10.1371/journal.pntd.0009540 (PMC8282061; doi:10.1371/journal.pntd.0009540)
Supplement: Attachment — Submitted filename: League et al._Responses to reviewer comments_PLOS NTDs.docx [file pntd.0009540.s014.docx]

| **Comment** | **Response** |
| --- | --- |
| **Reviewer 1** | |
| This indicates that there may be regional differences in selection pressures that lead to harmonic convergence. However, as the authors note, the two studies are not directly comparable due to differences in experimental design (competitive vs non-competitive matings), so it is difficult to draw firm conclusions in either direction. This inability to firmly reject or confirm previous findings is a major limitation of the work. The presence of other confounding effects such as artefacts of colonization further diminish the generalizability of the results, limiting impact. | We thank the reviewer for carefully parsing our results and agree with these comments on the limitations of our conclusions. We acknowledge these limitations in lines 369–380 and 461–472 so that our readers can carefully place our findings in the context of those of previous studies. We state in lines 432–436 that we cannot rule out potential artifacts resulting from the initial stages of laboratory colonization. Our study minimized these potential effects by using a low generation colony recently derived from dengue-endemic field sites. Therefore, our results are less susceptible to these effects than other studies employing mosquitoes that have been maintained in lab culture for many generations. We now suggest how future research might help to parse these potential influences on the traits we examined (lines 436–438). |
| The authors do not specify, but it appears that matings were given unlimited time. One possible advantage of harmonic convergence is to shorten the mating time, an advantage for females and their future offspring in the sense that they can more rapidly return to a safe harborage. However, in the absence of predators this advantage disappears. | In our harmonic convergence experiments, male and female pairs were given five minutes to form a copula. We have added this important piece of information to lines 166–167 of the revised manuscript:  “For both assays, males and females were given five minutes to court before starting a new trial with different individuals.”    We agree that time to mating is interesting and could be addressed in a separate study. Predation has been reported in *Anopheles* swarms (Yuval and Bouskila, 1993; PMID: 28313313), but is unlikely to be a significant pressure in *Ae. aegypti*, which swarms around the human host. However, disruption due to swatting, host defense, or movement could potentially impact *Ae. aegypti* mating. |
| An assumption of this work is that males can be accurately classified as “converging” or “non-converging” based on a single attempt with a randomly selected female. Is there evidence that males that display convergence will always display it, and vice versa? If there is sufficient variance in female flight tones, it is not unreasonable to think that males can better match some tones but not others. | The reviewer makes an interesting point. Harmonic convergence is likely not a binary, discrete trait, with some males always converging and others males never converging. Rather, it is more probable that some males are more likely to converge while others are less likely to converge. We also agree that female flight tones are an important source of variation.  There are several ways to assess the likelihood of males to converge. We selected a method in which males are given equal opportunity to perform harmonic convergence in a single trial. This method has been used in previous work in this field (for example: Cator and Harrington, 2011 and Reitmayer et al., 2020), We now discuss how these points might impact the interpretation of our results in lines 386–391 of the Discussion and suggest further experiments to help clarify these questions:  “An additional consideration is the robustness of harmonic convergence as a readout of male mating success across multiple male-female encounters. Similar to previous studies, our study design classifies males as either converging or non-converging based on a single male-female courtship interaction. However, the repeatability of convergence events both within the same courting pair and between combinations of individuals across multiple interactions merits further investigation.” |
| For measurement of flight traits, no indication is provided as to standardization of time of day, as flight behavior is strongly controlled by the circadian clock. | The Supporting information Methods states that “All parental recordings were performed between the hours of 09:00–18:00 and the temperature and humidity conditions were 26.47 ± 1.13˚C and 59.01 ± 7.95% RH for the reproductive fitness assays and 25 ± 1.8°C and 68.1 ± 2.0% RH for the flight performance assays.” We have moved this text from the Supporting information to lines 159–161 of the main text Methods. |
| Line 81: “Mating in Aedes mosquitoes occurs rapidly (<60 s) in aerial swarms [16] composed primarily of males and only a small number of females [17,18].” This statement is misleading and misrepresents what is given in the provided references. For example, in REF 17, the authors of that study describe that mating in Aedes can occur in swarms, but also in small groups or individually, citing evidence for each. | To capture this nuance, we have modified this sentence (now lines 84–86) to read:  “Mating in *Aedes* mosquitoes is rapid (<60 s) and most often occurs in aerial swarms composed primarily of males and only a small number of females [16], but also in small groups or individually [17,18].” |
| Lin147: “low generation” means what, exactly? The authors should be more precise here since this is a subjective term. | Generally speaking, a low generation line is one that is in lab culture for five or less generations, as defined in Aldersley et al., 2019. We have modified this sentence, now lines 149–152, to include this convention, as well as other clarifying details regarding the collection method of the Thai mosquitoes:  “All experiments were performed across two generations (F_2_ parents and their offspring for experiment one and F_3_ parents and their offspring for experiment two) using a low generation (i.e., five or less generations in the laboratory [43]) *Ae. aegypti* population originating from a single collection of eggs from Kamphaeng Phet Province, Thailand (16°27′ 48”N, 99°31′ 47”E).” |
| Lin150: experiments were conducted on adults 2-6 days post emergence. I worry about this range. 2-day old adults are likely not able to display full feeding/host seeking behaviors. This could be an additional source of confounding variation in the data. | We appreciate this concern. We have observed the full range of feeding and host-seeking behaviors using similarly aged Thai colony mosquitoes, which are annually supplemented with eggs from field-caught mosquitoes. We have added this information to lines 155–157 of the Methods as follows:  “All experiments were conducted on mosquito adults at 2–6 days post-emergence, as at this age range, related Thai females have reached reproductive maturity and display the full range of feeding and host-seeking behaviors [30].” |
| Lin160: Authors should describe what their criteria for “definitively rejected” were. The methods in their 2011 paper were much more precise, maybe cite that here if the same criteria were used. | We have modified this text (now lines 170–172 of the Methods), removing the subjective term, and have added the reference to Cator and Harrington, 2011, as our criteria for rejection were the same.  “The recording was terminated either after the female rejected the male or a mating copula was formed, as described previously [19].” |
| Lin163: please clarify whether “replicated twice” means “performed twice” or “performed three times” (an initial experiment and two replicate experiments). From the follow-up text, it appears the experiment was performed twice, which means it was replicated only once. | To clarify this, lines 175–178 of the Methods now read as follows:  “The experiment was performed twice for both reproductive fitness (119 total mating pairs: 59 pairs recorded in experiment one and 60 pairs in experiment two) and male flight performance experiments (84 total pairs: 42 pairs recorded in experiments one and two).”  Similar corrections were made to clarify this distinction elsewhere in the text (see red text throughout). |
| Lin177: after bloodmeal was any indication used to confirm that all mosquitoes fed to repletion? Differences in life history traits could be due to differences in the amount of blood imbibed. | Females were fed to repletion for at least five minutes and engorgement was verified by visually examining females after feeding. We have added this, as well as our institutional exemptions and approvals to perform these experiments, to lines 187–191 of the revised Methods:  “After this period, females were transferred to individual containers and provided access to a human host (co-authors SAP or LJC) until females had fed to repletion, which was verified by visual examination for engorgement (Cornell IRB Human Subjects Activity Exemption, FWA 00004513, and Imperial College London Health and Safety approved)." |
| Line 282: :another reference to “low generation” of colony | Resolved as mentioned above (see lines 149–152). |
| Fig 5-7. These figures are just a re-expression of the same data from Figs2-4, looking at total variation. These are not needed, as the data is already presented and the normality/non-normality/range of the data is already visible. The data is presented a 3rd time as Table 1. This can probably be moved to the supplement, as it does not add anything beyond what is already presented. | The data in Figs 2–4 is divided by convergence status, whereas the data in Figs 5–7 shows the raw data, regardless of convergence status. However, after additional consideration, we agree with the reviewer that Figs 5–7 and Table 1 contain considerable overlap with Figs 2–4 and that they are better suited for the Supporting information. Accordingly, Figs 5–7 and Table 1 have been redesignated as S2–S4 Figs and S2 Table, respectively, and their corresponding portions of the Results have been relocated to the S1 Supporting information Results (see lines 183–220 of the S1 Supporting information file). |
| Lin324-325. This drop in egg numbers between mothers and daughters is concerning. Even if the trait itself were not highly heritable, I would expect the overall range to be similar. This suggests problems in colonization which the authors allude to briefly in the discussion and make it difficult to generalize these data. | We agree with the reviewer that the lower egg laying of the daughters in our study was surprising. This trend was observed in both experiment trials. As the reviewer points out, we note these trends in what is now the S1 Supporting information Results lines 197–202 and we addressed possible explanations for this in lines 429–438 of the Discussion, adding a new portion on future experiments involving multiple colonization events and additional generations in the laboratory mentioned above and below to lines 436–438. Our methods were conducted such that mothers and daughters were reared and handled in the same manner (i.e., same density, diet, etc.), as explained in lines 148–157 of the Methods and lines 41–56 of the Supporting information Methods. The equivalency of these methods are supported by mother (2.98 ± 0.10 mm) and daughter (2.93 ± 0.10 mm) wing lengths, which despite being statistically different were nevertheless biologically comparable. We have added a note clarifying these points to lines 194–196 of the Methods and 197–201 of the Supporting information Results:  “Offspring from each pair were used in reproductive fitness and flight performance assays and were reared in individual families under the same density and diet conditions as parents (S1 Supporting information).”  “Consistent with previous observations in low and high generation colonies [38,40,62], both mother (SD=78.367) and daughter (35.984) first egg clutch size distributions were extremely variable (S2 Table) and differed significantly (KW test, P<0.001), perhaps due in part to constraints associated with using a low generation population of mosquitoes recently collected from the field.” |
| Line 442: The authors are commended for discussing the limitations of the study, but their phrasing “we cannot entirely rule out…” gives the impression that they can mostly rule these out, when in fact that is not the case. Ruling these out would require multiple colonization events and substantially more replication. Quite the opposite, as the small scale of this study (only two replicates over three generations) renders it particularly susceptible to the whims of these confounding effects. | We agree with this point, and have modified this text in lines 432–438 of the manuscript as follows:  “Conversely, in spite of our use of a low generation field-derived colony, we cannot rule out countervailing effects on variation from bottlenecks, founder effects, and the early stages of laboratory colony adaptation, which can eventually cause divergent reproductive phenotypes and reduced fitness and genetic diversity in mosquitoes [66–68,79]. Future work involving multiple colonization events examined over additional generations and biological replicates could help to resolve the contributions of these potential effects.” |
| **Reviewer 2** | |
| Line 191: Unclear. By having ‘, or’ are your describing the male fertility trials or describing a second type of assay? | We are defining insemination capacity. To clarify, we have modified this sentence (now lines 206–209) to read:  “After recording their courtship flight interaction with a female, we assessed male fertility by measuring father and son insemination capacity, defined here as the proportion of females a male inseminated out of the total number of females presented to him over the course of the assay (S1 Supporting information).” |
| Line 195: unclear. Was it a single male from each of 57 unique families? | We intended to convey that we tested a single male from each of the 57 unique families. We have modified the text (now lines 211–213) to make this clear:  “For fathers, a single male from each of the 57 unique families was tested, with 37 fathers tested in experiment one (13 converged and 24 non-converged) and 20 in experiment two (12 converged and eight non-converged).” |
| Line 230: I’m unclear about the appropriateness of the distributions in some of the models used. It seems as though LMMs were applied to count data which were not normally distributed for example, table 1 states that data for most assays were not normally distributed, but then figure 4/supp info states that LMMs were used for some of those assays. For example, to me “father/son total mating attempts” or “father/son total contacts” seem to be count data best modelled using GLMMs with Pois/negbin distributions not LM/LMMs. Is that not correct? Additionally, as your detailed explanations are far away in the supp info, perhaps adding the model type/distribution/link function information to somewhere more easily accessible up front like table 1 or I guess even the s1 tables may help readers understand exactly what models you ran for each assay and how appropriate they were. | We thank the reviewer for these suggestions regarding our statistical analyses. We did use linear mixed models that assume normality of the residuals. However, as most of the count responses had relatively large means, assuming normality is reasonable for our data and was verified by checking the residuals of the models in each case, as detailed in the Statistical methods section of the Supporting information Methods. Furthermore, the models we used are robust to these assumptions, as has been shown, for example, in Schielzeth et al., 2020 (doi: 10.1111/2041-210X.13434).  We agree that the main text Methods would benefit from statistical model details that were originally placed in the Supporting information Methods. We have added the relevant model details, and refer readers to tests of model assumptions, statistics, and raw numerical data, in lines 254–263 and 266–267 of the Methods:  “P-values <0.05 were considered statistically significant and were derived from minimal models, which included linear mixed effects models (LMMs) [50,51,58], generalized linear mixed models (GZLMM) [50], linear models (LM) [50], and generalized linear model (GZLM) [50] (for full model details, tests to verify model assumptions, and detailed model statistics, see S1 Supporting information and S1 Stats). A one-sample Kolmogorov-Smirnov (KS) and an independent samples Kruskal–Wallis (KW) test were used to characterize and compare parent and offspring reproductive fitness and flight performance parameter distributions. Kaplan-Meier curves [52,53,59] and a Cox frailty model [54,57] were used to visually compare and test for differences in daughter survival based on parental convergence status.”  “Raw numerical data for all figures and tables are included in S1 Dataset.” |
| Line 820: please provide speaker spec details | We have added detailed specs for the speakers we used for the flight performance assay to lines 75–78 of the Supporting information Methods:  “For the custom-built flight response cage, the speaker (Sony MDR-E9LPL 13.5 mm speaker; frequency: 18-22,000 Hz; sensitivity: 104 dB/mW) was suspended 8 cm below the lid of a 30 cm^3^ clear plastic cage and was attached to a timing belt (Synchroflex Timing Belt, 4660-950, Technobots, Rugby, UK) on top of the cage through a 0.5 cm x 22 cm track in the lid.” |
| Line 832: unclear. How long did it take the speaker to go from one side of the cage to the other? | The arm takes 0.48 s to move the 22 cm, and so moves at a rate of 0.458 m/s. We have corrected this and added the additional details to lines 86–89 of the Supporting information Methods:  “After this, the silent speaker began to move for an approximately 10 s acclimation period, with the arm taking 15 ms to accelerate to its 0.458 m/s (i.e., 0.48 s to move across the 22 cm track) back and forth speed across the cage.” |
| Line 838: ‘the through” typo | We thank the reviewer for catching this. This typo has been corrected (see line 94 of the Supporting information Methods). |
| Line 862: why would males not be successful at contacting the 200 RPM speaker when they could get stationary, 300 & 400 RPM do you think? | This is a great question. We think the male is getting information on the female’s velocity from her flight tone frequency. When there was too large of a discrepancy between the speed of the speaker and the flight tone frequency it emitted, the males did not track the speaker and respond phonotactically. However, as this explanation is still conjectural and would require additional testing before we are comfortable presenting it, we have not elaborated on this point in the revised manuscript and share this hypothesis here only for the reviewer’s sake. |
| Line 929 (S1 fig): I’m unclear about the camera in the set up. Did it swing back and forth at the same speed of the speaker on a separate timing belt? Is that what you mean by parallel to speaker movement? If it was to the side of the box as the line figure depicts how did it maintain focus? I would really love to see a photo of the entire unit if you have it rather than the line box drawing. | The camera was stationary and placed along the same horizontal axis of movement as the speaker. It was able to maintain focus on the speaker over the 22 cm distance it moved towards and away from it. We have edited S1 Fig to show a photo of the entire setup, rather than a diagram. We have also included a supplementary video with annotation to help clarify these points (S1 Video; see lines 241–242 of the revised Methods). Lines 234–237 and 723–725 of the manuscript and lines 110–112 of the Supporting information Methods now clarify these points. For example, lines 234–237 of the main text Methods now read as follows:  “All flight performance trials were video recorded using a stationary high-definition camera (DCR-SR68, Sony Corporation, New York, NY, USA) positioned along the same horizontal axis as the moving speaker at a distance that allowed the camera to maintain focus for the entirety of the speaker’s path (S1 Fig).” |
| Line 326: I’d insert wing length here rather than just size as we don’t know how you measured size until line 760. | This section was relocated to the Supporting information Results, and now follows references to wing lengths in the updated main text Methods (line 154) and the Supporting information Methods (lines 18–19). However, we have changed the text of lines 191–194 of the Supporting information Results (which precedes the reference cited by the reviewer here) to:  “Although father and son size as determined by wing length differed significantly in their distributions (KW test, P=0.041), fathers (2.21 ± 0.09 mm) and sons (2.16 ± 0.10 mm) were of comparable size and varied only moderately (S5 Fig A, B; S3 Table).” |
| Line 428: "measures measured" seems like an odd word choice. | We have corrected this sentence (lines 417–419 of the Discussion) as follows:  “We detected robust variation in the parent and offspring reproductive fitness and flight performance traits measured here (S1 Supporting information; S2–S4 Figs; S2 Table), suggesting ample variation for sexual selection to act upon.” |
| Line 464: Does your work support the idea that potentially “harmonic convergence is a by-product of the physical (e.g. aerodynamic) coordination” (Andres et al. 2020)? With all the words on harmonic convergence and sexual selection, I think that this alternate notion needs be addressed somewhere in your manuscript, if not the conclusion section. | The precise reasons for the difference between our current study and previous studies with respect to harmonic convergence are unclear. However, to address this interesting possibility, we have added the following to lines 381–386 of the Discussion in a new paragraph discussing alternate possibilities regarding the nature and role of harmonic convergence in mating interactions:  “In addition to potentially serving as a signal that females use to distinguish between males, harmonic convergence may occur as a consequence of the physical, aerodynamic coordination entailed in copula formation [20,69]. However, even if convergence is partly a byproduct of copula formation, our finding that males that successfully mate (i.e., inseminate females) do not sire offspring with higher mating success remains—converging males would still be more likely to form a mating copula than non-converging males.” |
| Abstract: I don’t think it is good practice to include the importance of findings of ‘previous work’ in the summary statement of your abstract. I’d suggest re-writing lines 39- and focus on the importance of the work specific to this study | We have modified this sentence in lines 44–47 of the Abstract (and in a similar sentence in lines 399–400 of the Discussion), which now reads:  “Together, our findings suggest that vector reproductive control programs should treat harmonic convergence as an indicator of some, but not all aspects of inherent quality, and that sexual selection likely affects *Ae. aegypti* in a trait-, population-, and environment-dependent manner.” |
| Line 94 felt a bit broad with the mention of nutritional gifts and parental effort. Are there examples of direct material benefits relating to mosquitoes. If not, maybe state this. If so, it would be interesting to learn about them here. | There are currently no examples of mosquito nuptial gifts as the term has been conventionally defined and understood, namely, as nutritional gifts or direct material benefits provided to the female by the male in conjunction with mating. If the term is construed more broadly, our lab has documented survival benefits triggered by mating in *Aedes aegypti* females (Villarreal et al., 2018). However, this effect is not due to a direct nutritional gift given at mating, but rather to downstream effects of male accessory gland proteins in females that we do not currently understand. Further, it has not been shown that this “gift” underlies female preference. We have added a brief reference to this information in lines 97–99 of the revised Introduction and have cited three studies in *Ae. aegypti* that discuss the topic in greater detail:  “In some cases, variation in direct material benefits offered by males to females, such as nutritional gifts or parental effort, underlie these preferences, although no examples of such direct benefits have been documented in mosquitoes [17,30,31].” |
| Line 103: should ‘as’ be ‘are’? | We thank the reviewer for pointing this out. This typo has been corrected (see line 107 of the Introduction). |
| **Reviewer 3** | |
| Flight performance assays were performed by testing each male only once in a 2-minute trial. This experiment assume that all males have the same motivation to locate a sound source. However, this might not be the case. If the variables used to assess flight performance are directly correlated to mating success, for instance, males should exhibit similar performance in several trials. If this is not the case, the experiment is affected by other factors, for instance, by how motivated individual males were during the trial. | To clarify, we did not employ the male flight performance assay as a measure of male mating success. While it is true that the ability to perceive, localize, and track moving sound sources is a key component of male mosquito mating behavior (this has been added to lines 298–299 of the Results to emphasize this point), the flight performance assay was intended to provide an additional measure of male quality (similar to the way that Reitmayer et al., 2020 examined immune performance; see lines 410–413 of the Discussion). In good genes models, traits are not necessarily related to male mating success (see lines 102–110 of the Introduction). Participation in the flight performance assay requires the coordinated integration of multiple sensory and physiological systems and thus provides information on aspects of male quality that the other fitness measures do not.  Inter-male variation across those attempts is shown in what are now Fig 4, S4 Fig, and S2 Table, and was used to test our hypotheses about harmonic convergence and heritability (Fig 4D, H). By recording male attempts, contacts, and success rates, we were able to capture the motivation aspect of the phonotactic response that the reviewer alludes to, but also the accuracy of those attempts and the aptitude of the male to successfully track and contact the speaker. We also controlled for genetic background, age, larval environment, and time of day in our experiments.  Males were provided many opportunities to engage the speaker within each two-minute trial (see S1 Video), and while assessment of intra-male variation within trials is not the subject of this study, we have provided raw flight performance data for individual males in S1 Dataset of the revised submission. |
| In addition, the experiment does not show if there are statistical differences on flight performance among males. I am not sure that the experimental design provides data to evaluate traits directly related to mating success. | These comments are addressed in the response to the first comment from Reviewer 3 above. |
| Finally, the authors acknowledge that unlike male fertility and female fecundity, flight performance has not been evaluated as a fitness trait in mosquitoes before. For these reasons, conclusions based on this trait that might be misleading. | Some of these comments are addressed above. We developed the flight performance assay to assess a performance trait separate from reproductive output. Our analysis shows that this type of performance was neither related to harmonic convergence nor heritable. |
| The large amount of work reflected by the results is remarkable.  Two main findings are presented in the manuscript. First, harmonic convergence does not signal male quality. Second, quality traits examined in this study displayed low heritability. The way results are presented to the reader make this section easy to follow. On the contrary, some figures are hard to read due to the big amount of information condensed in several panels which contain, in many cases, redundant titles and legends. Also, Table 1 does not provide useful information in the main text. I suggest moving that table to supplemental information. On the contrary showing that parental harmonic convergence does not signal offspring convergence is an important result as this finding challenges previous studies. I suggest including Supplemental Figure S3 in the manuscript. | We appreciate this comment from the reviewer. We agree that the figures contained some redundant information and have removed unnecessary titles and condensed the figure legends. We have also placed Table 1 (and its corresponding figures; see above) in the Supporting information as S2 Table in the revised manuscript. After further consideration, we also agree that S3 Fig represents an important finding for this area of research and have thus moved it into the main text Results as Fig 2E. We have updated that figure and its legend accordingly (see lines 286–288 of the Results). |
| The authors did a great job at making strong cases to support the main conclusions. However, I found the flight performance assay problematic. Evaluating flight performance as a trait directly linked to paternal mating success is innovative, however, the absence of replicates proving statistical evidence of differences among individuals might lead to incorrect conclusions. Based on the methods it is unclear if flight performance is associated with traits, like flight agility or reflexes, that directly correlate with mating success. Testing if a male shows consistent flight performance in several replicates would make a stronger case to support these conclusions. | These comments are addressed above. |
| Although most analyses limitations are acknowledged and discussed by the authors, I find concerning the use of tethered females to test sexual selection. Free-flying females react to approaching males by modulating their flight-tones (1,2). Mosquito courtship is therefore a complex acoustic interaction affected by mosquito motion. Harmonic convergence assays, however, were performed by using “semi-tethered” females. Although this approach has been used in the past to evaluate harmonic convergence and it allows some flight mobility, the female’s response is still constrained. This limitation might affect selection and should be acknowledged by the authors. 1. Simões PM V. et al. 2016 A role for acoustic distortion in novel rapid frequency modulation behaviour in free-flying male mosquitoes. J. Exp. Biol. 219, 2039–2047 | The benefits of the tethering technique employed in our study are mentioned in lines 26–33 of the Supporting information Methods. Further, the stereotypic female startle flight response to males during courtship is similar in tethered and free-flying females. We did not use this technique to test sexual selection, but only as an accommodation to tractably detect harmonic convergence between courting couples with precision. However, we now acknowledge the limitations mentioned in this comment in lines 391–395 of the Discussion in a new paragraph discussing harmonic convergence. Building on previous work involving free-flying individuals such as Simões et al., 2016, we also propose analysis of free-flying couples as a goal for future work on this subject:  “Finally, despite the advantages of examining harmonic convergence between free-flying males and tethered females [19,20,64], future studies building on previous work examining individual free-flying mosquito acoustics [70] could capture nuances of this behavior in free-flying couples that are not fully recapitulated using current standard methods.” |
| The authors found that father harmonic convergence does not signal son ability to converge. This finding is relevant as challenges previous studies and promote future research. Although it is acknowledged that the specific reason for this difference is unclear, the authors speculate that differences in selective pressures might led to differences in mating behavior between strains used in this and previous studies. Were differences on behavior detected? Is it possible to support this hypothesis using collected data? | We do not have high generation colony fitness data to directly compare with the low generation colony data produced in our experiments, which would require an entirely separate large-scale study on laboratory adaptation, founder effects, population differences, etc. arising over numerous generations in the lab (See lines 432–438 of the Discussion). Thus, we have cited numerous studies in lines 367–380 of the Discussion that provide our readers with reference data to provide context for interpreting our results. |
| In Figure 1, it is necessary to differentiate harmonic convergence from the traits that were tested as indicators of quality. As it is, Figure 1 can be misinterpreted. Using different geometric figures or labels to what shapes and colors mean will make the figure more intelligible. | Thank you for these thoughts. We agree that these suggestions would improve the figure and reduce potential misunderstandings. We have implemented these suggested changes in Fig 1 of the revise manuscript. |
| In Figures 2, 3 and 4, labels are redundant. For instance, the authors could add only one label in the figure to indicate convergence for all the panels. | These comments are addressed above. |
| Increase letter size in Figure 3. | We have increased the letter size in all figures to 12-point font. |
| The X axis of panels E and F are hard to read in Figure 7. Please increase space between numbers. | We have increased the font size of and spacing between the axes number labels in panels E and F of what is now S4 Fig, as well as in all other comparable figure panels. |
| Increase resolution of all figures. | We have reproduced our figures utilizing the PACE digital diagnostic tool to confirm that they now meet journal resolution requirements. |
| My main concern about this manuscript is that flight performance assays might not capture what the authors intended. The idea is very creative and might promote future research. However, proving that the flight performance assays capture traits directly associated with mating success is necessary. Would it be possible to perform a short experiment testing flight performance of the same males during two or three days? If not, it would be necessary to acknowledge this limitation in the discussion or explaining why the authors predicted that males will exhibit the same flight performance during harmonic convergence or insemination assays. | These comments are addressed above. See also lines 386–391 of the Discussion, which were added in response to similar comments regarding harmonic convergence from Reviewer 1. |
| A secondary concern is that figures condense a large amount of data, which in some cases makes them difficult to read. In addition, many figures contain redundant information. Reducing the number of labels and titles can make the figures clearer for the reader. Also, improving the resolution, size letter and space between numbers in the axis will make the manuscript more attractive and intelligible for a general audience. | These comments are addressed above. |
